# Investigations on the Key Odorants Contributing to the Aroma of Children Soy Sauce by Molecular Sensory Science Approaches

**DOI:** 10.3390/foods10071492

**Published:** 2021-06-28

**Authors:** Jia Huang, Haitao Chen, Zhimin Zhang, Yuping Liu, Binshan Liu, Baoguo Sun

**Affiliations:** 1 School of Light Industry, Beijing Technology and Business University, Beijing 100048, China; 15501111590@163.com (J.H.); 2030301028@st.btbu.edu.cn (Z.Z.); 1930201009@st.btbu.edu.cn (B.L.); sunbg@btbu.edu.cn (B.S.); 2 Beijing Advanced Innovation Center for Food Nutrition and Human Health, Beijing Technology and Business University, Beijing 100048, China; 3 Beijing Key Laboratory of Flavor Chemistry, Beijing Technology and Business University, Beijing 100048, China

**Keywords:** children soy sauce, gas chromatography-olfactometry, AEDA, FD factor, quantitative measurements, OAV, key odorants

## Abstract

To investigate the key odor-active compounds in children’s soy sauce (CSS), volatile components were extracted by means of solvent extraction coupled with solvent-assisted flavor evaporation (SE-SAFE) and solid-phase microextraction (SPME). Using gas chromatography-olfactometry (GC-O) and gas chromatography-mass spectrometry (GC-MS), we identified a total of 55 odor-active compounds in six CSSs by comparing the odor characteristics, MS data, and retention indices with those of authentic compounds. Applying aroma extract dilution analysis (AEDA), we measured flavor dilution (FD) factors in SE-SAFE isolates, ranging from 1 to 4096, and in SPME isolates, ranging from 1 to 800. Twenty-eight odorants with higher FD factors and GC-MS responses were quantitated using the internal standard curve method. According to their quantitated results and thresholds in water, their odor activity values (OAVs) were calculated. On the basis of the OAV results, 27 odorants with OAVs ≥ 1 were determined as key odorants in six CSSs. These had previously been reported as key odorants in general soy sauce (GSS), so it was concluded that the key odorants in CSS are the same as those in GSS.

## 1. Introduction

Soy sauce (SS) originated in China about 2700 years ago [1]. As a kind of condiment, SS was mainly manufactured in Asian countries, but it was consumed in various places around the world. In recent years, with the rapid development of children’s food, many children’s soy sauces (CSSs) have been supplied in the Chinese market. These CSSs are claimed to have more nutritional elements, to be manufactured by a special process, and to be more suitable for consumption by children; their prices are much higher than those of general SS (GSS). Odor is one of the important sensory properties of CSS; to our knowledge, there have been no reports to date on the flavor constituents of CSS, nor is there a Chinese standard for CSS.

To date, reports about the flavor constituents of SS have focused on GSS. From 1887, researchers began to investigate the volatile compounds in SS [2], and to date, there have been many reports about the volatiles in SS [3,4,5,6,7,8,9,10]. Among the volatile compounds identified, not all of them contribute to the overall odor profiles of SS. Gas chromatography-olfactometry (GC-O) analysis has been used as an effective method to screen the odor-active compounds from the volatiles in food extracts. Volatile components in Korean SS were extracted via solid phase microextraction (SPME) and solvent extraction, and the extracts were analyzed using GC-O. Eleven odor-active compounds were identified, and methional, 3-methylbutanoic acid, guaiacol, 2,5-dimethyl-4-hydroxy-3(2H)furanone (DMHF) and 2-ethyl-4-hydroxy -5-methyl-3(2H)furanone (HEMF) were found to have higher flavor dilution (FD) factors [3]. The key aroma compounds in Japanese SS were characterized using molecular sensory science approaches for the first time in 2007. Twenty-eight aroma-active compounds were identified by means of GC-O analysis in an isolate obtained from Japanese SS through solvent extraction combined with solvent-assisted flavor evaporation (SE-SAFE), and 13 compounds with odor activity values (OAVs) > 1 were determined to be the key odorants [4]. To clarify the compounds’ contributions to the odor profiles of Japanese SS, researchers from Japan have investigated the aroma compounds in SS by means of GC-O, and more than 60 aroma-active compounds have been identified. Among those odorants, some compounds, including guaiacol, 4-ethyl guaiacol, 2(and 3)-methylbutanal, methional, DMHF, HEMF, etc., have a higher detection frequency in the analyzed samples [5,6,7]. Odor components in Chinese SS have also been examined by means of GC-O, and more than 50 aroma-active compounds have been determined. Some substances, such as 2-phenylethanol, 3-methylbutanol, 3-methylbutanoic acid, 2(and 3)-methylbutanal, methional, benzeneacetaldehyde, HEMF and dimethyl trisulfide, have been identified as aroma-active compounds in all Chinese SS samples [8,9]. The odorants in five Chinese high-salt liquid-state soy sauces were investigated using modified gas chromatography-mass spectrometry-olfactometry. A total of 195 odor-active compounds were detected, and methional, maltol, guaiacol, 4-ethylguaiacol, 2-acetylpyrrole, 2-acetylfuran, 2-phenylethanol, furfural and DMHF showed high FD factors [10].

Because of the lack of reports about the odor-active compounds and key odorants in CSS, the aims of the present study were (i) to screen and identify the aroma-active compounds in CSS using GC-O, (ii) to quantitate the odorants identified, (iii) to identify the key odorants contributing to the characteristic odor of CSS by calculating the odor activity values (OAV, the ratio of an odorant concentration to its odor threshold) of those odor-active substances, and (iv) to determine if there are difference between CSS and GSS in key odorants.

## 2. Materials and Methods

### 2.1. Samples

Three Chinese children’s soy sauce samples (C1, C2, C3) were purchased from local supermarkets (Merry Mart and Yonghui superstores in Beijing, China); three Japanese children’s soy sauce samples (J1, J2, J3) were bought from online stores. The raw materials of samples were as follows. C1: water, organic defatted soybean, organic wheat and salt. C2: water, non-transgenic defatted soybean, wheat, corn, salt, sodium glutamate, disodium 5′-ribonucleotide, yeast extract, potassium sorbate, potassium acetylsulfonate and sucralose. C3: water, soybean, wheat flour, salt, sucrose, sodium glutamate and spices. J1: organic cabbage, organic common onion, organic radish, organic taro roots, organic pumpkin, organic scallop, organic soy sauce, natural Kombu and bonito. J2: soy sauce, powder of Kombu root, bonito, iron pyrophosphate and fructose syrup (from soybean and wheat). J3: non-transgenic soybean, wheat, salt, Kombu, extracts of Kombu, ethanol and vitamin B1. These samples were kept in a 4 °C refrigerator until extraction experiments were conducted.

### 2.2. Chemicals

Ethyl acetate (99.5%), 3-methylbutanal (99%), 2,3-butanedione (98%), 2,3-pentanedione (97%), ethyl 2-methylbutanoate (99%), 3-methylbutanol (99%), 1-octen-3-one (97%), 2,5-dimethylpyrazine (99%), 2,6-dimethylpyrazine (98%), 2-ethylpyrazine (98%), dimethyl trisulfide (98%), 2-ethyl-5-methylpyrazine (98%), nonanal (95%), 2,3,5-trimethylpyrazine (99%), propionic acid (99%), linalool (98%), ethyl 3-acetylpropionate (98%), 2-methylpropionic acid (99%), butanoic acid (99%), 3-methylbutanoic acid (99%), 2-furanmethanol (98%), methionol (98%), pentanoic acid (99%), ethyl phenylacetate (99%), 4-methylpentanoic acid (99%), methylcyclopentenolone (99%), guaiacol (99%), maltol (99%), 4-ethylguaiacol (98%) and 2-octanol (99 %, internal standard) were purchased from J&K Chemical Ltd. (Beijing, China). 2-methylbutanal (98%), ethyl propanoate (99.5%), ethyl 3-methylbutanoate (99%), hexanal (97%), octanal (99%), methional (98%), 2-ethyl-3,5-dimethylpyrazine (99%), 3-methyl-2-isobutyl pyrazine (>98%), benzeneacetaldehyde (95%), phenethyl alcohol (99%) and vanillin (>98%) were bought from Macklin Biochemical Co., Ltd. (Shanghai, China). Ethyl butanoate (>98%), ethyl 2-hydroxy-4-methylpentanoate (98%), (E,Z)-2,6-nonadienal (>95%), 4-ethylphenol (>97%) and 2,6-dimethoxyphenol (99%) were obtained from TCI (Shanghai, China). Ethyl 2-methylpropanoate (>98%), 2,3-diethyl-5-methylpyrazine (>98%), 2-acetylpyrazine (>98%), 3-methylpentanoic acid (>98%) and γ-dodecalactone (>98%) were supplied by Adamas reagent Co., Ltd. (Shanghai, China). Ethanol (>99%), acetic acid (>99%), anhydrous sodium sulfate and dichloromethane were purchased from Sinopharm Chemical Reagent Co., Ltd. (Beijing, China). Methanethiol (2000 μg/mL in toluene), 4-hydroxy-2,5-dimethyl-3(2H)-furanone (98%), 5-ethyl-4-hydroxy-2-methyl-3(2H)-furanone (97%) and phenylacetic acid (95%) were supplied by AccuStandard (New Haven, CT, USA), Aladdin Reagents (Shanghai, China) Co., Ltd., Ark Pharm Inc. (Chicago, IL, USA), Key Organics (Cornwall, England), respectively. C6-C28 normal alkanes were bought from Aldrich Chemical Co., Ltd. (Shanghai, China). Dichloromethane was freshly distilled prior to experiments.

### 2.3. Isolation of Volatiles from CSS

#### 2.3.1. SE-SAFE for Volatile Components in CSS

CSS samples (100 mL) were extracted with redistilled dichloromethane (50 mL × 3) at room temperature by stirring vigorously for 1.5 h × 3, and the obtained extracts were merged together. The volatiles were isolated from the combined extracts via high vacuum distillation using SAFE (Edwards TIC Pumping Station from BOC Edwards, England). The extract containing neutral and basic volatile components was obtained by washing the distillate from SAFE with 0.05 mol/L sodium carbonate solution (100 mL × 2) and saturated sodium chloride (50 mL × 3), respectively. The alkaline aqueous phase was acidified to a pH value of 2 using 0.5 mol/L HCl solution, and then the mixture was extracted with dichloromethane (50 mL × 3) to obtain the isolate containing acidic volatile compounds [4]. Both extracts were dried over anhydrous sodium sulfate for about 12 h and concentrated to approximately 3–5 mL with Vigreux columns (50 cm × 1 cm) (Beijing Jingxing Glassware Co., Ltd., Beijing, China) at 45 °C, and then they were further concentrated to 0.3 mL using gentle nitrogen streams. These concentrates were used for GC-O and GC-MS analyses.

#### 2.3.2. SPME for Volatile Constituents in CSS

The volatile compounds in CSS were also extracted by means of SPME, as described previously with some modifications [11]. A 2-cm (coated with 50/30 μm DVB/CAR/PDMS) SPME fiber (Supelco, Bellefonte, PA, USA) was preconditioned before extraction experiments in accordance with the manufacturer’s instructions. A mixture of 16 mL CSS and 2 g sodium chloride was placed in a 40-mL static headspace amber glass bottle fitted with a stir bar and a polytetrafluoroethylene (PTFE)-faced silicon septum. The extraction conditions for SPME obtained by optimizing experiments were as follows: equilibrium and extraction temperatures of 45 °C, an equilibrium time of 20 min, and an extraction time of 40 min. After the extraction experiment, the fiber was transferred to the injector port of GC for a 5-min desorption at 250 °C to conduct the GC-O and GC-MS analyses.

### 2.4. Analysis of Odor-Active Compounds in CSSs

#### 2.4.1. GC-O Analysis

GC-O was performed by means of an Agilent 7890 GC combined with an olfactory detection port (ODP3, Gerstel, Germany) and an FID (Agilent Technologies, USA). The GC effluent at the end of the capillary column was split into a 1:2 ratio by volume using a Y-type splitter and two uncoated deactivated fused silica capillaries between the FID and ODP. To maintain the nose sensitivity, the sniffing port was coupled with humidified air. The temperatures of the GC injector port, the FID, the transfer line of ODP3 and the olfactory port were 250 °C, 280 °C, 250 °C and 220 °C, respectively. The extracts were analyzed on both a DB-Wax column and a Hp-5MS column (Agilent, both are 30 m × 0.25 mm × 0.25 μm). When the DB-Wax column was used, the oven temperature was held at 40 °C for 2 min, increased to 80 °C at a rate of 8 °C/min, increased to 100 °C at a rate of 4 °C/min, then rose to 230 °C at a rate of 6 °C/min, and finally held at 230 °C for 5 min. When the Hp-5MS column was used, the oven temperature was held at 40 °C for 2 min, increased to 100 °C at a rate of 4 °C/min, ramped to 230 °C at a rate of 10 °C/min and finally held at 230 °C for 5 min. Ultra-high purity helium was used as the GC carrier gas at a constant flow rate of 1 mL/min. All concentrated fractions (1 µL) or SPME isolates were injected in splitless mode. During GC-O analyses, three trained evaluators (two females and one male, who had been trained to sniff the aromas of reference compound solutions with different concentrations in the laboratory for at least 3 months) from Beijing Key Laboratory of Flavor Chemistry at Beijing Technology and Business University sniffed the odors of the effluent from the sniffing port. When evaluators detected the odor, they needed to record the retention time (RT) and the odor characteristics. Analyses were carried out three times by each evaluator.

#### 2.4.2. GC-MS Analysis

GC-MS analyses for identification were conducted with an Agilent 7890B GC connected to an Agilent 5975 mass selective detector. The parameters, columns and temperature program for GC were the same as those employed in the GC-O analyses described above. Mass spectra in election ionization mode at 70 eV were recorded at 150 °C; the ion source temperature was kept at 230 °C. Detection was carried out in full-scan mode, and mass range was from 33 to 350 amu.

#### 2.4.3. Odor-Active Compound Identification

A series of normal alkanes were analyzed using GC-O and GC-MS under the conditions described in Section 2.4.1 and 2.4.2, and RTs of normal alkanes were measured. Retention indexes (RIs) of the detected odor-active compounds were computed on the basis of their RTs and the RTs of normal alkanes. If the concentrations of odor-active compounds were higher than the detection limits of the mass selective detector, their MS data were obtained, and they were positively identified by comparing their MS data, RIs and odor characteristics with those of standard compounds and data in NIST2014. If the concentrations of odor-active compounds were lower than the detection limits of the mass selective detector, and their MS data were not available, they were positively identified by comparing their RIs and odor characteristics with those of standard compounds.

### 2.5. Aroma Extract Dilution Analysis (AEDA)

For AEDA, CSS volatile extracts obtained by SE-SAFE were diluted stepwise with redistilled dichloromethane to obtain serial dilutions of 1:2, 1:4, 1:8, 1:16, 1:32, 1:64, …, and 1:4096 [4]. For SPME isolates, the dilution was carried out by changing the split ratio to 1:5, 1:10, 1:25, 1:50, 1:100, 1:200, 1:400, 1:600 and 1:800 [8]. All dilutions were subjected to GC-O analyses on a DB-Wax column under the conditions described in Section 2.4.1 until no odorant could be detected. The flavor dilution (FD) factor of every odorant was defined as the maximum dilution in which the odor compound could be detected by the evaluator. If FD factors from three evaluators were different, the highest FD factors were adopted.

### 2.6. Quantitation of Selected Odor-Active Compounds in CSS

The odor-active compounds giving peaks in GC-MS chromatograms and having FD factors ≥32 in SE-SAFE isolates or FD factors ≥25 in SPME extracts were quantitated using the internal standard curve method; 2-octanol was used as an internal standard. Firstly, a series of solutions of the mixture of internal standard and authentic compounds were prepared and analyzed by GC-MS under the conditions described in Section 2.4.2 except that selective ion monitoring mode was used. The standard curves were obtained by plotting the ratios of the peak areas of the authentic compounds relative to that of 2-octanol against their concentration ratios. Then 2-octanol (300 μL, 37.15 μg/mL) was added into 100 mL CSS, and its final concentration was 111.45 μg/L. The volatiles in CSS were extracted via SE-SAFE according to the method described in Section 2.3.1; the extracts were concentrated to 1 mL and analyzed by GC-MS. Finally, the concentrations of selected odor-active compounds in CSS were calculated on the basis of GC-MS analysis results and standard curves.

## 3. Results and Discussion

### 3.1. Odor Evaluation

SE-SAFE and SPME were used for isolating the volatile constituents from CSS. In order to confirm if the odorants contributing to the characteristic odor of CSS had been extracted, the odors of the isolates obtained were evaluated by three well-experienced evaluators. The results showed that both the liquid extract obtained by SE-SAFE and the fiber of SPME had the same overall aroma profile as CSS. They had caramel, cooked potato, smoky, sour and floral notes. The odor intensity of isolates obtained via SE-SAFE was stronger than that of SPME fiber. That is, the extraction methods used were appropriate.

### 3.2. Odor-Active Compounds Detected Using GC-O

The volatile isolates of six CSSs obtained via SE-SAFE and SPME were analyzed by means of GC-O; the odor-active regions were detected. To identify the structures of the odor-active compounds, their odor characteristics, mass spectra data and RIs were compared with the data obtained from the published literature and authentic standards. The results are listed in Table 1.

A total of 55 aroma-active compounds were identified from six CSSs on the DB-Wax and HP-5 columns in Table 1, including 10 esters, nine carboxylic acid, nine pyrazines, seven aldehydes, seven ketones, five alcohols, four phenols and four sulfur-containing compounds. Of 55 compounds, six odorants (1, 4, 5, 12, 27 and 41) were only identified in SPME isolates; most of them had lower boiling points. Meanwhile, eight odorants (20, 29, 31, 33, 35, 40, 47 and 53) were only identified in SE-SAFE isolates, and they had higher boiling points. The number of odorants identified in C1, C2, C3, J1, J2 and J3 were 44, 45, 43, 40, 45 and 44, respectively; there were 33 compounds in common for the six CSSs.

Ten ester compounds (2, 5, 6, 8, 10, 11, 28, 33, 41 and 53) were detected as odor-active compounds in six CSSs. Of the 10 ester compounds, nine esters were ethyl esters. All of them had been identified as volatile compounds in Chinese SS [9,12], Japanese SS [7], Thai SS [13] or Korean SS [14,15]; most of them had also been identified as odor-active compounds in SS; for example, ethyl propanoate, ethyl 2-methylpropanoate, ethyl butanoate, ethyl 2-methylbutanoate, ethyl 3-methylbutanoate and ethyl phenylacetate had been found in Japanese SS as aroma-active compounds [7,16]; ethyl acetate and ethyl propanoate had been identified as odorants in Chinese SS [8]. However, as odor-active compounds in SS, γ-dodecalactone and ethyl 2-hydroxy-4-methylpentanoate (EHMP) had not been reported. As SS volatiles, γ-dodecalactone was only identified in SS manufactured using *Bacillus* species and fused yeast [15], and EHMP only in Chinese SS [12] by MS. EHMP was a very important flavor compound; it occurred in fresh fruits, grape brandies, wines, etc. When this ethyl ester was mixed with C4−C10 alkanoic acids, it could enhance natural, ripe and tropical fruit flavors. It may have contributed greatly to the fruity odor of SS [17]. All of the esters identified were thought to be a result of two pathways. The first was the metabolism of yeasts. In the production process of SS, a variety of microorganisms, including yeast, lactic acid bacteria, *Aspergillus oryzae*, etc., were used. During SS fermentation, some esters were formed enzymatically through the metabolism of yeasts. The second pathway was the reaction of alkanol with organic acid during sterilization and storage; because the reaction was non-enzymatic catalysis, the reaction rate was slow, and the number of esters formed was less. The production of esters depended on many factors, such as aeration, concentrations of organic acids, alcohols and their precursors, etc. [18].

Nine carboxylic acids (23, 29, 31, 35, 37, 40, 42, 43 and 55), including four linear-chain carboxylic acids, four branched-chain carboxylic acids and one aromatic acid, were identified as odorants in six CSSs; acetic acid, butanoic acid, 3-methylbutanoic acid and 3-methylpentanoic acid were the common substances in six samples. All of these organic acids have been reported as volatiles and odor-active compounds of GSS in the published literature [4,9,10,14], and they were formed as microorganism metabolic products. For example, the metabolism of lactic acid bacteria led to the production of acetic acid, propionic acid, butanoic acid, etc. [19]. The precursors of 2-methylpropionic acid, 3-methylbutanoic acid and phenylacetic acid were valine, leucine and phenylalanine, respectively; these acids could be produced as yeast metabolic products by transamination and decarboxylation oxidation [20].

Nine pyrazines (16, 17, 18, 20, 22, 25, 26, 27 and 34) were also detected as flavor compounds in six CSSs; among them, neither 3-methyl-2-isobutylpyrazine (27) nor 2-acetylpyrazine (34) had been identified in GSS as volatiles and odor-active compounds. 3-methyl-2-isobutylpyrazine was only detected in the C2 sample, and 2-acetylpyrazine was found in six samples. These pyrazine compounds could be formed by three pathways. Firstly, they might originate from the raw materials of SS, including roasted wheat and wheat bran, which contained pyrazine compounds, such as 2-methylpyrazine, 2,6-dimethylpyrazine, 2-ethyl-5-methylpyrazine, etc. [21,22]. Secondly, they were formed by a Maillard reaction during processing; their precursors were α-amino acids, carbohydrates, and α-dicarbonyl compounds. Soybean was an important material for producing SS; it contained oil and 18 free α-amino acids [23]. Soybean was roasted under heating before being used; oil in soybean could yield α-dicarbonyl compounds upon oxidation [24]; the wheat contained carbohydrates. These substances were conducive to the Maillard reaction. Thirdly, some pyrazines were among the microbial metabolic products; for example, under the same fermentation conditions, some pyrazines, including 2,5-dimethylpyrazine, 2,6-dimethylpyrazine, 2-ethyl-5-methylpyrazine, 2,3,5-trimethylpyrazine and 2-ethyl-3,5-dimethylpyraizine, were identified as volatile constituents of the solid-state fermentation product of bacteria, but they were not detected in the solid-state fermentation product of yeast [25]. Tetramethylpyrazine could be synthesized by *Bacillus subtilis* through the multi-step bioconversion of glucose to acetoin as a precursor [26].

Seven aldehydes (3, 12, 14, 21, 32, 36 and 54) were identified as odor-active compounds. 2(3)-methylbutanal and benzeneacetaldehyde belonged to Strecker aldehyde. Not only could they be formed through the Strecker degradation of isoleucine (or leucine) and phenylalanine, but also were derived from the corresponding amino acid catabolism by the Ehrlich pathway [20]. Hexanal, octanal, nonanal and (E,Z)-2,6-nonadienal were lipid-derived compounds. Soybean seeds contained more than 20% soybean oil, which contained monounsaturated and polysaturated fatty acids, such as oleic acid, linoleic acid, arachidonic acid, etc. [27]. These four aliphatic aldehydes could be derived from unsaturated fatty acid (UFA) through an oxidation reaction. Vanillin could be produced by microorganisms, such as bacteria, fungi, yeast or engineered microbial cells; its precursor was ferulic acid, present in the cell wall of wheat (6.6 g/kg), which was one of the materials of SS, or lignin, which exists in soybeans and wheat. The bioconversion of ferulic acid into vanillin occurs in both aerobic and anaerobic conditions [28].

Seven ketones (7, 9, 15, 44, 47, 49 and 50) were identified as odor-active compounds; all of them have been found in GSS. There were two main pathways for the formation of 2,3-butanedione and 2,3-pentanedione. The first was that they were generated during the Maillard reaction. 2,3-butanedione was formed through the sugar degradation pathway, and its precursor was glucose. 2,3-pentanedione was produced by the sugar degradation pathway and through the further interaction of sugar degradation products with amino acids, and its precursors were glucose and L-alanine [29]. The second pathway was yeast fermentation. 2,3-butanedione was formed by decomposition of the α-acetolactic acid synthesized by yeast, and 2,3-pentanedione from α-aceto-α-hydroxybutyric acid [30]. 1-octen-3-one, belonging to the lipid-derived compound, was formed via the autoxidation of UFAs [31]. The formation of both methylcyclopentenolone and maltol were associated with the Maillard reaction. Methylcyclopentenolone has been identified in volatile compounds of the glucose-tyrosine model system and the glucose-histidine model system [32], and maltol has been formed directly from the Amadori product which was the intermediate of the Maillard reaction [33]. Both DMHF and HEMF could be produced not only by the Maillard reaction but also could be biosynthesized by yeasts [2].

There were five alcohols (4, 13, 30, 38 and 46) identified as odor-active compounds. Ethanol, 3-methylbutanol and phenethyl alcohol were the metabolites of yeast; ethanol was formed by the EMP pathway and both 3-methylbutanol and phenethyl alcohol were derived from amino acid catabolism via the Ehrlich pathway [20]. 2-furanmethanol was a known thermal degradation product of ribose during the Maillard reaction. Linalool was identified in Japanese CSS, though not in Chinese CSS. It might come from the kombu, which is only used in Japanese CSS, because some kombu contains linalool [34].

Four phenols (45, 48, 51 and 52) were detected as aroma-active compounds; they could be formed by two pathways. Firstly, they were synthesized by different yeasts from some phenolic acids present in materials used for manufacturing SS, for example, 4-ethylphenol from *p*-coumaric acid and 4-ethylguaiacol from ferulic acid [35]. Secondly, they were produced by lignin pyrolysis; for instance, guaiacol and 2,6-dimethoxyphenol could be obtained from coconut shell pyrolysis [36]. Before wheat and soybeans were used for manufacturing SS, they were roasted. Lignin underwent pyrolysis, and some phenols were produced.

Four sulfur-containing compounds (1, 19, 24 and 39) were identified as odor-active compounds, and they were the common odorants in six CSS samples. Methanethiol was only detected in the isolate obtained via SPME; because its boiling point was about 6 °C, it was removed easily when the isolate obtained by solvent extraction was concentrated to recover the solvent. It arose from the degradation of methionine or cysteine derivatives. Dimethyl trisulfide came from the oxidation of methanethiol. Methional was a Strecker aldehyde, and it could originate from Strecker or microbiological degradation of methionine. Methionol was formed through the decarboxlation of 4-methylthio-2-oxobutyric acid, which was transamination product of methionine [20,37]. These four sulfur-containing compounds have been found in GSS.

### 3.3. The FD Factor of Odor-Active Compounds in Six CSSs

To screen more important odor-active compounds from 55 odorants identified in six CSSs, their FD factors were measured via GC-O, combined with AEDA. The results obtained are listed in Table 2.

Based on Table 2, it can be seen that 4-ethylguaiacol (burnt, smoky) had the highest FD factor of all the CSS isolates obtained by both SE-SAFE (FD factor = 4096) and SPME (FD factor = 800). Both 3-methylbutanoic acid (sweaty, cheese-like) and DMHF (caramel-like) possessed the highest FD factor (4096) among all the extracts obtained by SE-SAFE; both methional (cooked potato) and guaiacol (burnt, smoky) possessed the highest FD factor (800) among all the isolates obtained by SPME. Aside from the compounds mentioned above, some odor-active substances, such as 2-ethyl-3,5-dimethylpyraizine (roasty, earthy), benzeneacetaldehyde (honey-like), phenethyl alcohol (floral), HEMF (caramel-like), 2,6-dimethoxyphenol (burnt, smoky), etc., also had higher FD factors in either SE-SAFE extracts or SPME isolates. These compounds might cause the six CSS samples to possess some common odor characteristics. There were also some odorants which had the highest FD factor only in one sample. For example, dimethyl trisulfide had a higher FD factor (sulfur/cabbage, FD factor = 1024) only in J3, EHMP (fruity, FD factor = 2048) only in C1, methionol (cooked potato, FD factor = 1024) only in J3, maltol (caramel-like, FD factor = 1024) only in C1, vanillin (vanilla, FD factor = 1024) only in C2 and phenylacetic acid (honey, FD factor = 1024) only in C1. These odorants resulted in the odor differences among the six CSSs. Most of the RIs of these compounds with higher FD factors on the DB-Wax column were more than 1400. They were likely to have contributed the most to the overall aroma profile of CSS.

### 3.4. Quantitation of the Odor-Active Compounds with FD Factors ≥32 or 50

To calculate OAVs, a total of 28 compounds with FD factors ≥32 (in SE-SAFE isolates) or ≥25 (in SPME isolates) were quantitated by constructing standard curves; the results gained are shown in Table 3 and Table 4.

Of the 28 odor-active compounds, acetic acid had the highest concentration (57,948–406,726 μg/L) in all CSSs; the result was similar to Wang’s data relating to odorants in GSS [10]. Four odorants, including ethyl butanoate, ethyl 2-methylbutanoate, ethyl 3-methylbutanoate and octanal, had lower concentrations in six CSSs, and their values were less than 1 μg/L.

The odorants quantitated could be grouped into eight categories according to their chemical structures, that is, alcohols, carboxylic acids, esters, aldehydes, ketones, phenols, pyrazines and sulfur-containing compounds. Among these eight categories, the total concentrations of carboxylic acids in all of six CSSs were higher than those of the other seven categories, and the values ranged from 62,711 μg/L to 413,936 μg/L. The value of the total concentrations of all the quantitated odorants in J3 (542,622 μg/L) was the highest, and that in J1 (74,373 μg/L) was the lowest.

Of the six CSSs, the total concentrations of ketones (61,452 μg/L) and esters (58.69 μg/L) were the highest in C1; those of alcohols (45,928 μg/L), phenols (4884 μg/L) and pyrazines (582 μg/L) were the highest in C3; and carboxylic acids (413,936 μg/L), sulfur-containing compounds (25,775 μg/L) and aldehydes (25,368 μg/L) had their highest concentrations in J3.

C2 contained the lowest concentrations of both sulfur-containing compounds (2029 μg/L) and aldehydes (593 μg/L). In J1, the concentrations of carboxylic acids (62,711 μg/L), alcohols (1694 μg/L), ketones (4638 μg/L), phenols (408 μg/L) and esters (19.05 μg/L) were the lowest among the six samples. The lowest concentration of pyrazines (115 μg/L) was found in J2. These results showed that there were great differences in the concentrations of odor-active compounds among the six samples.

### 3.5. OAVs of Odor-Active Compounds in Six CSSs

To evaluate further the contributions of the 28 odor-active compounds to the aromas of the six CSSs and to screen for the key odorants, their OAVs were calculated based on their obtained concentrations and odor detection thresholds in water, and the results are shown in Table 5.

Of the 28 odor-active compounds, 27 odorants in some CSSs yielded OAVs ≥ 1, and their OAVs were vastly different. Only octanal had an OAV < 1 in all six CSSs; it did not contribute to the odors. The number of odorants with OAVs ≥1 in C1, C2, C3, J1, J2 and J3 was 26, 21, 27, 22, 23 and 25, respectively. In most samples, methional, 3-methylbutanal, 2-methylbutanal, HEMF, guaiacol and benzeneacetaldehyde had higher OAVs than the other odor-active compounds; they contributed the most to the overall odor profile and imparted cooked potato, malty, caramel-like, smoky and honey-like odors to the six CSSs, and these odors also comprise the characteristic notes of GSS. In the six samples, there were much bigger differences among the OAVs of methionol (OAVs = 20–583), 2-ethyl-3,5-dimethylpyrazine (OAVs = 7–263), 4-ethylguaiacol (OAVs = 10–136), 3-methylbutanol (OAVs = 2–74), DMHF (OAVs = 6–342), dimethyl trisulfide (OAVs = 31–317), 4-ethylphenol (OAVs = 1–30) and ethyl 3-methylbutanoate (OAVs = 2–34); these odorants caused the six CSSs to have some different notes. The OAVs of the other odorants were close; these odorants had similar contributions to the odors of six CSSs.

According to the OAV results, 27 odorants identified in different CSSs were further screened as key odorants contributing to the characteristic aroma of CSS. Except for EHMP, the other odor-active compounds had been identified as key odorants of GSS. Therefore, according to the results obtained, it was concluded that the key odorants of CSS should be same as those of GSS. The question of whether CSSs contain more nutritional components requires further study.

## 4. Conclusions

In summary, this study provides the comprehensive determination of the key odorants of six CSSs. A total of 55 aroma-active compounds were positively identified by comparing their MS data, RIs and odor characteristics with those of standard compounds, and their FD factors were measured using GC-O, coupled with AEDA. Twenty-seven volatile compounds with OAVs ≥ 1 were furtherly screened as key odorants contributing to the characteristic aroma profile of six CSSs by means of quantitative analyses combined with the calculation of OAVs. The results show that the key odorants in CSS were the same as those in GSS. Further research should focus on how to quantitate the odorants with higher FD factors and without responses to MS detection, as well as performing aroma reconstitution experiments and omission tests to further confirm the results and investigating if there are differences between the nutrients of CSS and GSS.

## Figures and Tables

**Table 1:** Odor-active compounds identified in six CSS samples.

No.	Compound	RI		Odor Quality	Chinese CSSs			Japanese CSSs			Isolate ^c^	Identification ^d^
		DB-Wax ^a^	HP-5 ^b^		C1	C2	C3	J1	J2	J3		
1	methanethiol	690	<600	sulfur, garlic	+	+	+	+	+	+	S	O, RI, S
2	ethyl acetate	880	- ^e^	fruity	-	-	-	-	-	+	A,S	O, RI, S
3	2(3)-methylbutanal	919	651	malty	+	+	+	+	+	+	NB,S	O, MS, RI, S
4	ethanol	930	<600	alcoholic	+	-	+	-	+	+	S	O, RI, S
5	ethyl propanoate	939	- ^e^	fruity	-	-	-	+	+	-	S	O, RI, S
6	ethyl 2-methylpropanoate	964	750	fruity	+	+	+	+	+	+	A,NB,S	O, MS, RI, S
7	2,3-butanedione	973	603	butter	+	+	+	+	+	+	A,NB,S	O, RI, S
8	ethyl butanoate	1048	800	fruity	+	+	+	+	+	+	A,S	O, MS, RI, S
9	2,3-pentanedione	1055	- ^e^	butter	-	-	+	+	+	+	NB,S	O, RI, S
10	ethyl 2-methylbutanoate	1061	839	fruity	+	+	+	+	+	+	A,NB,S	O, MS, RI, S
11	ethyl 3-methylbutanoate	1072	847	fruity	+	+	+	+	+	+	A,NB,S	O, MS, RI, S
12	hexanal	1090	795	green	-	-	-	+	+	+	S	O, RI, S
13	3-methylbutanol	1205	- ^e^	malty	+	+	+	+	+	+	NB,S	O, MS, RI, S
14	octanal	1284	1005	fatty, green	+	+	+	+	+	+	A,NB,S	O, MS, RI, S
15	1-octen-3-one	1297	983	mushroom-like	+	+	+	+	+	+	A,NB,S	O, RI, S
16	2,5-dimethylpyrazine	1314	- ^e^	roasty	-	-	-	+	+	+	NB,S	O, RI, S
17	2,6-dimethylpyrazine	1332	912	roasty	+	+	+	-	+	+	NB,S	O, MS, RI, S
18	2-ethylpyrazine	1339	- ^e^	roasty	+	+	+	+	+	+	NB,S	O, RI, S
19	dimethyl trisulfide	1383	980	sulfur, cabbage	+	+	+	+	+	+	A,NB,S	O, MS, RI, S
20	2-ethyl-5-methylpyrazine	1386	- ^e^	roasty, nutty	-	+	-	-	-	-	NB	O, RI, S
21	nonanal	1390	1090	fatty	-	+	+	-	+	-	NB,S	O, MS, RI, S
22	2,3,5-trimethylpyrazine	1404	998	roasty, earthy	+	+	+	+	+	+	NB,S	O, MS, RI, S
23	acetic acid	1440	660	sour	+	+	+	+	+	+	A,S	O, MS, RI, S
24	methional	1450	911	cooked potato	+	+	+	+	+	+	A,NB,S	O, MS, RI, S
25	2-ethyl-3,5-dimethylpyrazine	1461	1078	roasty, earthy	+	+	+	+	+	+	A,NB,S	O, MS, RI, S
26	2,3-diethyl-5-methylpyrazine	1492	- ^e^	earthy	+	+	+	-	-	-	NB,S	O, RI, S
27	3-methyl-2-isobutyl pyrazine	1500	- ^e^	green	-	+	-	-	-	-	S	O, RI, S
28	ethyl 2-hydroxy-4-methylpentanoate	1530	1068	fruity	+	+	+	-	-	-	NB,S	O, MS, RI, S
29	propionic acid	1533	- ^e^	sour	+	-	-	-	-	-	A	O, RI, S
30	linalool	1547	1106	green, woody	-	-	-	+	+	+	NB,S	O, RI, S
31	2-methylpropionic acid	1564	- ^e^	sour	+	+	+	-	-	-	A	O, RI, S
32	(E,Z)-2,6-nonadienal	1579	- ^e^	cucumber	+	+	+	+	+	+	NB,S	O, RI, S
33	ethyl 3-acetylpropionate	1603	1020	fruity	+	-	-	-	-	-	NB	O, RI, S
34	2-acetylpyrazine	1625	1025	bready, roasty	+	+	+	+	+	+	NB,S	O, RI, S
35	butanoic acid	1629	793	sour	+	+	+	+	+	+	A	O, MS, RI, S
36	benzeneacetaldehyde	1637	1045	honey-like	+	+	+	+	+	+	A,NB,S	O, MS, RI, S
37	3-methylbutanoic acid	1663	870	sweaty, cheese	+	+	+	+	+	+	A,S	O, MS, RI, S
38	2-furanmethanol	1668	860	coffee, nutty	+	+	+	+	+	+	NB,S	O, MS, RI, S
39	methionol	1712	990	cooked potato	+	+	+	+	+	+	NB,S	O, MS, RI, S
40	pentanoic acid	1731	900	sour	-	+	-	-	+	-	A	O, RI, S
41	ethyl phenylacetate	1781	1260	floral	+	-	-	-	+	+	S	O, MS, RI, S
42	3-methylpentanoic acid	1788	- ^e^	sweaty, cheese	+	+	+	+	+	+	A,S	O, RI, S
43	4-methylpentanoic acid	1791	- ^e^	sweaty, cheese	+	+	-	+	+	+	A,S	O, RI, S
44	methylcyclopentenolone	1827	1030	caramel-like	+	+	+	+	-	+	A,NB	O, RI, S
45	guaiacol	1855	1082	burnt, smoky	+	+	+	+	+	+	A,NB,S	O, MS, RI, S
46	phenethyl alcohol	1909	1110	floral	+	+	+	+	+	+	A,NB,S	O, MS, RI, S
47	maltol	1969	1113	caramel-like	+	+	+	+	+	+	A	O, MS, RI, S
48	4-ethylguaiacol	2026	1280	burnt, smoky	+	+	+	+	+	+	NB,S	O, MS, RI, S
49	4-hydroxy-2,5-dimethyl-3(2H)-furanone	2033	1075	caramel-like	+	+	+	+	+	+	A,NB,S	O, MS, RI, S
50	5-ethyl-4-hydroxy-2-methyl-3(2H)-furanone	2058	1136	caramel-like	+	+	+	+	+	+	A,NB,S	O, MS, RI, S
51	4-ethylphenol	2169	1165	smoky	+	+	+	+	+	+	A,NB,S	O, MS, RI, S
52	2,6-dimethoxyphenol	2264	- ^e^	burnt, smoky	+	+	+	+	+	+	A,NB,S	O, MS, RI, S
53	γ-dodecalactone	2382	- ^e^	fatty	-	+	+	-	-	-	NB	O, MS, RI, S
54	vanillin	2570	1398	vanilla	+	+	+	+	+	+	A,NB,S	O, RI, S
55	phenylacetic acid	2578	- ^e^	honey	+	+	+	-	+	+	A,S	O, MS, RI, S

^a^ Retention index of compounds on a DB-WAX column. ^b^ Retention index of compounds on a HP-5 column. ^c^ Isolate: S indicates compounds isolated by solid-phase microextraction; NB represents compounds isolated from the neutral-basic volatile fraction of the extract obtained by SE-SAFE; A represents compounds isolated from the acidic volatile components of the extract obtained by SE-SAFE. ^d^ Identification methods: O means confirmed by odor characteristics; MS refers to identification by comparison with the NIST 2014 mass spectra database; RI means confirmed by retention index; S means confirmed by authentic standards. ^e^ indicates that the compound was not isolated by the HP-5 column. + means the compound was identified in the sample; -means the compound is not identified in the sample.

**Table 2:** FD factors of odor-active compounds in six CSS samples.

No.	Compounds	FD Factor ^a^											
		Chinese CSS Samples						Japanese CSS Samples					
		C1		C2		C3		J1		J2		J3	
		SAFE	SPME	SAFE	SPME	SAFE	SPME	SAFE	SPME	SAFE	SPME	SAFE	SPME
1	methanethiol	-	10	-	50	-	10	-	50	-	50	-	5
2	ethyl acetate	-	-	-	-	-	-	-	-	-	-	128	1
3	2(3)-methylbutanal	64	10	32	1	64	5	64	100	32	25	256	10
4	ethanol	-	25	-	-	-	25	-	-	-	25	-	5
5	ethyl propanoate	-	-	-	-	-	-	-	1	-	5	-	-
6	ethyl 2-methylpropanoate	2	100	-	1	-	10	-	10	-	25	-	10
7	2,3-butanedione	64	5	16	50	32	1	16	25	32	10	32	5
8	ethyl butanoate	-	200	-	5	16	50	32	-	32	1	4	1
9	2,3-pentanedione	-	-	-	-	16	-	-	1	-	5	4	-
10	ethyl 2-methylbutanoate	256	100	-	25	128	50	-	10	-	50	-	50
11	ethyl 3-methylbutanoate	512	-	8	-	128	-	16	10	8	50	32	50
12	hexanal	-	-	-	-	-	-	-	5	-	5	-	1
13	3-methylbutanol	32	1	4	5	2	5	1	-	32	5	32	5
14	octanal	32	1	32	10	32	10	4	1	4	10	64	-
15	1-octen-3-one	32	25	8	100	32	25	512	10	32	25	512	5
16	2,5-dimethylpyrazine	-	-	-	-	-	-	1	10	4	5	4	10
17	2,6-dimethylpyrazine	1	-	1	-	2	-	-	-	16	-	2	5
18	2-ethylpyrazine	-	5	128	10	256	10	32	1	256	25	256	1
19	dimethyl trisulfide	64	400	64	50	64	50	256	200	64	50	1024	10
20	2-ethyl-5-methylpyrazine	-	-	4	-	-	-	-	-	-	-	-	-
21	nonanal	-	-	-	10	1	-	-	-	2	-	-	-
22	2,3,5-trimethylpyrazine	64	5	64	100	128	10	32	1	32	-	32	1
23	acetic acid	8	5	2	5	4	25	2	-	4	25	8	10
24	methional	4096	800	512	800	256	800	4096	800	4096	800	4096	800
25	2-ethyl-3,5-dimethylpyrazine	1024	100	512	600	1024	400	256	100	256	200	512	1
26	2,3-diethyl-5-methylpyrazine	-	100	256	-	-	200	-	-	-	-	-	-
27	3-methyl-2-isobutylpyrazine	-	-	-	5	-	-	-	-	-	-	-	-
28	ethyl 2-hydroxy- 4-methylpentanoate	2048	400	16	50	32	5	-	-	-	-	-	-
29	propionic acid	2	-	-	-	-	-	-	-	-	-	-	-
30	linalool	-	-	-	-	-	-	1	5	4	1	-	1
31	2-methylpropionic acid	4	-	2	-	8	-	-	-	-	-	-	-
32	(E,Z)-2,6-nonadienal	2	1	1	1	2	1	1	10	2	10	16	-
33	ethyl 3-acetylpropionate	8	-	-	-	-	-	-	-	-	-	-	-
34	2-acetylpyrazine	32	800	256	50	32	800	32	100	32	50	8	200
35	butanoic acid	16	-	16	-	512	-	16	-	4	-	16	-
36	benzeneacetaldehyde	1024	10	256	25	512	50	256	200	512	200	1024	200
37	3-methylbutanoic acid	4096	25	4096	200	4096	400	4096	100	4096	100	4096	10
38	2-furanmethanol	64	50	4	100	64	400	4	400	4	100	8	400
39	methionol	512	25	128	10	256	10	64	200	128	1	1024	10
40	pentanoic acid	-	-	16	-	-	-	-	-	4	-	-	-
41	ethyl phenylacetate	-	10	-	-	-	-	-	-	-	1	-	1
42	3-methylpentanoic acid	32	1	256	5	8	5	8	1	8	10	8	1
43	4-methylpentanoic acid	32	5	8	5	-	-	8	1	4	-	4	1
44	methylcyclopentenolone	2	-	2	-	2	-	64	-	-	-	256	-
45	guaiacol	2048	800	1024	800	4096	800	1024	800	1024	800	2048	800
46	phenethyl alcohol	4096	800	2048	800	4096	400	512	100	4096	200	4096	800
47	maltol	1024	-	32	-	32	-	16	-	32	-	32	-
48	4-ethylguaiacol	4096	800	4096	800	4096	800	4096	800	4096	800	4096	800
49	4-hydroxy-2,5-dimethyl-3(2H)-furanone	4096	800	4096	200	4096	800	4096	800	4096	800	4096	800
50	5-ethyl-4-hydroxy-2-methyl-3(2H)-furanone	4096	800	1024	5	1024	5	4096	800	4096	800	4096	800
51	4-ethylphenol	64	400	8	50	32	200	4	10	16	50	32	100
52	2,6-dimethoxyphenol	512	-	512	1	1024	50	256	-	512	-	512	-
53	γ-dodecalactone	-	-	2	-	2	-	-	-	-	-	-	-
54	vanillin	256	1	1024	-	32	5	256	25	-	50	128	400
55	phenylacetic acid	1024	1	512	-	512	-	-	-	64	-	128	-

^a^ FD factor, flavor dilution factor, determined on a DB-Wax column. -means the compound is not identified in the isolate.

**Table 3:** Standard curves of 28 odor-active compounds quantitated in six CSS samples.

No.	Compound	Quantified Ion	Standard Curves	R^2^
3	2-methylbutanal	57	y = 0.0069x + 1.4039	0.999
3	3-methylbutanal	71	y = 0.0027x + 1.0098	0.996
6	ethyl 2-methylpropanoate	71	y = 0.1264x + 0.0185	0.998
8	ethyl butanoate	71	y = 0.6002x−0.0036	0.995
10	ethyl 2-methylbutanoate	102	y = 0.7420x−0.0164	0.995
11	ethyl 3-methylbutanoate	88	y = 0.5337x−0.0065	0.994
13	3-methylbutanol	55	y = 0.0016x−0.0079	0.999
14	octanal	84	y = 0.3571x + 0.0137	0.991
19	dimethyl trisulfide	126	y = 1.0809x−0.1085	0.998
22	2,3,5-trimethylpyrazine	122	y = 0.0449x−0.6999	0.995
23	acetic acid	60	y = 0.0002x−1.0603	0.993
24	methional	48	y = 0.0006x + 0.0061	0.997
25	2-ethyl-3,5-dimethylpyrazine	135	y = 0.2299x−0.1852	0.994
28	ethyl 2-hydroxy-4-methylpentanoate	69	y = 0.0425x−0.1146	0.993
35	butanoic acid	60	y = 0.0017x−0.6759	0.990
36	benzeneacetaldehyde	91	y = 0.0299x−1.0006	0.998
37	3-methylbutanoic acid	73	y = 0.0034x−0.8599	0.994
38	2-furanmethanol	98	y = 0.0005x + 0.01316	0.994
39	methionol	106	y =0.0003x + 0.0056	0.999
45	guaiacol	109	y = 0.0618x + 0.0339	0.999
46	phenethyl alcohol	91	y = 0.0297x−0.0239	0.992
47	maltol	126	y = 0.4418x−0.4070	0.998
48	4-ethylguaiacol	137	y = 0.4116x + 0.2833	0.994
49	4-hydroxy-2,5-dimethyl-3(2H)-furanone	128	y = 0.1502x + 0.4608	0.992
50	5-ethyl-4-hydroxy-2-methyl-3(2H)-furanone	125	y = 0.0780x + 0.2168	0.991
51	4-ethylphenol	107	y = 0.1818x−0.0239	0.997
52	2,6-dimethoxyphenol	154	y = 0.0016x−0.0140	0.998
55	phenylacetic acid	91	y = 0.4581x + 1.6573	0.998

**Table 4:** Concentrations of 28 odor-active compounds in six CSS samples.

No.	Compound	Conc. (μg/L) ^a^					
		Chinese CSS Samples			Japanese CSS Samples		
		C1	C2	C3	J1	J2	J3
	carboxylic acids						
23	acetic acid	207266 ± 16707	60588 ± 773	215125 ± 17321	57948 ± 4717	143403 ± 5401	406726 ± 4688
35	butanoic acid	2844 ± 23	2917 ± 43	17069 ± 1522	2712 ± 8	-	2842 ± 160
37	3-methylbutanoic acid	3037 ± 289	2190 ± 64	14999 ± 971	1922 ± 37	2072 ± 37	3028 ± 215
55	phenylacetic acid	6335 ± 507	2336 ± 118	20620 ± 434	129 ± 16	731 ± 90	1340 ± 49
	Total	219482 ± 17526	68031 ± 998	267813 ± 20248	62711 ± 4778	146206 ± 5528	413936 ± 5112
	alcohols						
13	3-methylbutanol	16360 ± 224	3424 ± 124	2090 ± 74	437 ± 27	3256 ± 114	18144 ± 1296
38	2-furanmethanol	19735 ± 1162	6839 ± 29	40182 ± 4350	833 ± 69	6014 ± 381	16780 ± 1319
46	phenethyl alcohol	5089 ± 337	1627 ± 65	3656 ± 232	424 ± 23	2328 ± 240	5955 ± 631
	Total	41184 ± 1723	11890 ± 218	45928 ± 4656	1694 ± 119	11598 ± 735	40879 ± 3246
	ketones						
47	maltol	18116 ± 942	6672 ± 68	7265 ± 803	1428 ± 127	3711 ± 392	6173 ± 107
49	4-hydroxy-2,5-dimethyl-3(2H)-furanone	2352 ± 117	8904 ± 91	13676 ± 50	223 ± 5	460 ± 80	576 ± 40
50	5-ethyl-4-hydroxy-2-methyl-3(2H)-furanone	40984 ± 1673	1774 ± 46	1091 ± 116	2987 ± 30	9009 ± 541	28771 ± 752
	Total	61452 ± 2732	17350 ± 205	22032 ± 969	4638 ± 162	13180 ± 1013	35520 ± 899
	sulfur-containing compounds						
19	dimethyl trisulfide	0.46 ± 0.03	0.31 ± 0.01	0.27 ± 0.01	2.20 ± 0.02	0.47 ± 0.01	3.14 ± 0.28
24	methional	2652 ± 166	154 ± 6	143 ± 16	1377 ± 22	1663 ± 82	4801 ± 392
39	methionol	9907 ± 618	1875 ± 222	3448 ± 298	731 ± 71	2371 ± 260	20971 ± 1186
	Total	12559 ± 784	2029 ± 228	3591 ± 314	2110 ± 93	4034 ± 342	25775 ± 1578
	aldehydes						
3	2-methylbutanal	3545 ± 87	86 ± 7	1242 ± 105	865 ± 16	1291 ± 43	10337 ± 1005
3	3-methylbutanal	1739 ± 97	189 ± 15	785 ± 16	1256 ± 117	1234 ± 85	10571 ± 511
14	octanal	0.55 ± 0.05	0.54 ± 0.04	0.48 ± 0.02	0.20 ± 0.01	0.31 ± 0.01	0.96 ± 0.05
36	benzeneacetaldehyde	1206 ± 83	317 ± 28	981 ± 74	548 ± 27	704 ± 28	4459 ± 65
	Total	6491 ± 267	593 ± 50	3008 ± 195	2669 ± 160	3229 ± 156	25368 ± 1581
	phenols						
45	guaiacol	245 ± 19	146 ± 4	1249 ± 37	82.62 ± 2.62	101 ± 4	293 ± 13
48	4-ethylguaiacol	599 ± 8	109 ± 10	305 ± 7	95.10 ± 0.18	42.09 ± 2.02	81.80 ± 5.92
51	4-ethylphenol	388 ± 25	16.14 ± 0.38	338±15	12.01 ± 0.96	20.03 ± 1.39	37.53 ± 1.04
52	2,6-dimethoxyphenol	518 ± 16	500 ± 52	2992 ± 56	218 ± 11	559 ± 52	521 ± 35
	Total	1750 ± 68	771 ± 66	4884 ± 115	408 ± 15	722 ± 59	933 ± 55
	pyrazines						
22	2,3,5-trimethylpyrazine	144 ± 10	198 ± 2	540 ± 16	123 ± 11	113 ± 1	157 ± 8
25	2-ethyl-3,5-dimethylpyrazine	32.98 ± 1.53	6.53 ± 0.52	42.02 ± 3.63	1.08 ± 0.03	2.03 ± 0.12	15.19 ± 0.78
	Total	177 ± 12	205 ± 3	582 ± 20	124 ± 11	115 ± 1	172 ± 9
	esters						
6	ethyl 2-methylpropanoate	2.66 ± 0.07	-	0.35 ± 0.02	-	1.34 ± 0.10	-
8	ethyl butanoate	0.33 ± 0.01	0.09 ± 0.01	0.94 ± 0.06	0.07 ± 0.01	0.11 ± 0.01	0.42 ± 0.04
10	ethyl 2-methylbutanoate	0.52 ± 0.05	-	0.15 ± 0.00	0.07 ± 0.00	0.44 ± 0.04	0.49 ± 0.04
11	ethyl 3-methylbutanoate	0.66 ± 0.00	-	0.27 ± 0.02	0.05 ± 0.00	0.53 ± 0.03	0.77 ± 0.06
28	ethyl 2-hydroxy-4-methylpentanoate	54.52 ± 4.88	19.23 ± 0.07	28.62 ± 1.71	18.86 ± 0.26	22.47 ± 0.69	37.37 ± 1.09
	Total	58.69 ± 5.01	19.32 ± 0.08	30.33 ± 1.81	19.05 ± 0.27	24.89 ± 0.87	39.05 ± 1.23
	All total	343154 ± 23117	100888 ± 1768	347868 ± 26519	74373 ± 5338	179109 ± 7835	542622 ± 12481

^a^ Average concentrations of triplicate experiments.

**Table 5:** OAVs of 28 aroma compounds in six CSS samples.

No.	Compound	DOT (μg/L)	OAV ^f^					
			Chinese CSS Samples		Japanese CSS Samples			
			C1	C2	C3	J1	J2	J3
24	methional	0.43 ^a^	6166	359	332	3202	3867	11165
3	3-methylbutanal	0.50 ^a^	3479	379	1570	2512	2468	21142
3	2-methylbutanal	1.5 ^a^	2363	58	828	576	861	6891
50	5-ethyl-4-hydroxy-2-methyl-3(2H)-furanone	20 ^b^	2049	89	55	149	450	1439
45	guaiacol	0.84 ^a^	292	174	1487	98	120	349
39	methionol	36 ^a^	275	52	96	20	66	583
36	benzeneacetaldehyde	5.2 ^c^	232	61	189	105	135	858
25	2-ethyl-3,5-dimethylpyrazine	0.16 ^b^	206	41	263	7	13	95
48	4-ethylguaiacol	4.4 ^a^	136	25	69	22	10	19
13	3-methylbutanol	220 ^a^	74	16	9	2	15	82
49	4-hydroxy-2,5-dimethyl-3(2H)-furanone	40 ^a^	59	223	342	6	11	14
19	dimethyl trisulfide	0.0099 ^a^	46	31	27	222	47	317
10	ethyl 2-methylbutanoate	0.013 ^a^	40	-	12	5	34	38
46	phenethyl alcohol	140 ^a^	36	12	26	3	17	43
51	4-ethylphenol	13 ^a^	30	1	26	1	2	3
6	ethyl 2-methylpropanoate	0.089 ^a^	30	-	4	-	15	-
11	ethyl 3-methylbutanoate	0.023 ^a^	29	-	12	2	23	34
52	2,6-dimethoxyphenol	29 ^c^	18	17	103	8	19	18
38	2-furanmethanol	1900 ^d^	10	4	21	<1	3	9
47	maltol	2500 ^d^	7	3	3	1	2	3
22	2,3,5-trimethylpyrazine	23 ^d^	6	9	24	5	5	7
37	3-methylbutanoic acid	490 ^a^	6	4	31	4	4	6
23	acetic acid	99000 ^a^	2	1	2	1	1	4
35	butanoic acid	2400 ^a^	1	1	7	1	<1	1
55	phenylacetic acid	6100 ^a^	1	<1	3	<1	<1	<1
28	ethyl 2-hydroxy-4-methylpentanoate	55 ^e^	1	<1	1	<1	<1	1
8	ethyl butanoate	0.76 ^a^	<1	<1	1	<1	<1	1
14	octanal	3.4 ^a^	<1	<1	<1	<1	<1	<1

^a^ Odor thresholds in water according to Czerny et al. [38]. ^b^ Odor thresholds in water according to Semmelroch and Grosch [39].^c^ Odor thresholds in water according to Mall and Schieberle [40]. ^d^ Odor thresholds in water according to Buttery et al. [41]. ^e^ Odor thresholds in water according to Lytra et al. [17]. ^f^ Odor activity value (ratio of the concentration to the odor threshold).

## Data Availability

The data shown in this study are contained within the article.
